# Oxygen and an Extracellular Phase Transition Independently Control Central Regulatory Genes and Conidiogenesis in *Aspergillus fumigatus*

**DOI:** 10.1371/journal.pone.0074805

**Published:** 2013-09-05

**Authors:** Myoung-Hwan Chi, Kelly D. Craven

**Affiliations:** Plant Biology Division, Samuel Roberts Noble Foundation, Ardmore, Oklahoma, United States of America; Yonsei University, Korea, Republic Of

## Abstract

Conidiogenesis is the primary process for asexual reproduction in filamentous fungi. As the conidia resulting from the conidiogenesis process are primarily disseminated via air currents and/or water, an outstanding question has been how fungi recognize aerial environments suitable for conidial development. In this study, we documented the somewhat complex development of the conidia-bearing structures, termed conidiophores, from several *Aspergillus* species in a subsurface (gel-phase) layer of solid media. A subset of the isolates studied was able to develop conidiophores in a gel-phase environment, but exposure to the aeriform environment was required for the terminal developmental transition from phialide cells to conidia. The remaining Aspergilli could not initiate the conidiogenesis process until they were exposed to the aeriform environment. Our observations of conidiophore development in high or low oxygen conditions in both aeriform and gel-phase environments revealed that oxygen and the aeriform state are positive environmental factors for inducing conidiogenesis in most of the aspergilli tested in this study. Transcriptional analysis using *A. fumigatus* strain AF293 confined to either the aeriform or gel-phase environments revealed that expression of a key regulatory gene for conidiophore development (*AfubrlA*) is facilitated by oxygen while expression of another regulatory gene controlling conidia formation from phialides (*AfuabaA*) was repressed regardless of oxygen levels in the gel-embedded environment. Furthermore, by comparing the developmental behavior of conidiation-defective mutants lacking genes controlling various regulatory checkpoints throughout the conidiogenesis pathway, we propose that this aerial response by the fungus requires both oxygen and the phase transition (solid to aeriform), with these environmental signals integrating into the upstream regulatory pathway and central regulatory pathway of conidiogenesis, respectively. Our findings provide not only novel insight into how fungi respond to an aerial environment to trigger development for airborne conidia production but also the relationship between environmental factors and conidiogenesis regulation in aspergilli.

## Introduction

Fungal species belonging to the genus *Aspergillus* (collectively called the aspergilli) are some of the most common and widespread saprophytic and/or pathogenic fungi in nature, occupying an extraordinarily diverse range of ecological niches. One of the main reasons for such ubiquity is the prolific production of uninucleated, hydrophobic airborne conidia via a process known as conidiogenesis [1]. The dispersal of aspergilli conidia is essential for both short and long distance spread of the fungus, and in many cases directly impacts human affairs; as a source of allergens, mycotoxins and even human disease [2], [3]. Understanding fungal conidiogenesis is an attractive topic not only because of the practical importance but it also provides insight into how eukaryotic cells interact with the extracellular environment to regulate their development. Yet to date, the majority of genetic research on conidiogenesis in the aspergilli has been conducted on the model species, *Aspergillus nidulans* [4], [5], [6].

The conidiogenesis process among aspergilli begins with aerial stalks emerging from branching thick-walled hyphae known as foot cells [5], [7]. At the end distal from the subtending foot cell, the stalk swells isotropically into a vesicle. Subsequently, a layer of specialized cells termed metulae bud from each vesicle. From each metulae arise two or three phialides, budding from the tip and then differentiating into spore-producing (conidiogenous) cells [5], [7], [8]. Most aspergilli follow a similar developmental program, although properties such as conidiophore size, density and color vary according to the species. Comparison of such developmental differences among species is key to identifying unique characteristics that may be related to their distinct lifestyle.

Mechanistically, the production of airborne spores typically occurs in the aboveground parts of the fungus, where they can be efficiently disseminated to initiate new colonies. Indeed, the phase transition from a solid media to an aerial environment is a well-known trigger for the induction of conidiogenesis in aspergilli, but it has remained unclear how the fungus recognizes this environmental shift.

A number of genes have been identified that regulate the sequence of events involved in the critical cellular differentiation processes associated with conidiogenesis in *A. nidulans* [5], [9]. Such studies have revealed central regulatory components including *brlA*, *abaA*, and *wetA* that control precise spatial and temporal expression of other genes specific for distinct stages of conidiogenesis [5], [10], [11], [12], [13], [14]. Upstream activators including *fluG*, *flbA*, *flbB*, *flbC*, *flbD* and *flbE* have been shown to be required for proper activation of *brlA* and conidiogenesis [15], [16], [17], [18], [19], [20], [21], [22], while downstream *velvet* regulators including *vosA*, *veA*, *velB*, and *velC* are required for feedback regulation of *blrA* and secondary metabolism [9], [23], [24], [25], [26].

Comparative studies between *A. nidulans* (Ani) and *A.fumigatus* (Afu) have revealed that the genes belonging to the central and downstream regulatory pathway controlling conidiogenesis are quite well-conserved between the two species in terms of developmental function and regulatory mechanisms [27], [28], [29]. However, significant differences between the model and the pathogenic fungus have been found through functional analysis of the upstream regulators. For example, the mutant lacking Flbs or FluG in *A. nidulans* was almost completely blocked in conidiation whereas the same mutants in *A. fumigatus* (strain AF293) showed a delayed/reduced phenotype (*in ▵AfuflbA*, *▵AfuflbB*, and *▵AfuflbE* [22], [27], [30]) or were identical to wild type (in *▵AfufluG* [27]) in an air-exposed (aeriform) environment. Why such a difference exists in the phenotypic intensity of the mutants lacking these upstream regulators between the two fungi remains an unsettled question. Interestingly, the phenotype of the *A. fumigatus* upstream regulator mutants was intensified when they were tested in a liquid-submerged environment, in which *A. fumigatus* (strain AF293) produces conidia while *A. nidulans* remains in a vegetative hyphal phase [22], [27], [30]. This led us to hypothesize that there are fundamental differences between the two fungi, whereby the former recognizes and responds to distinct environments for conidiogenesis, which may directly stimulate the central regulatory pathway.

In this study, we found that both oxygen and the aeriform (In this study, ‘aeriform’ is defined as gas-phase representing *physical* status of the air, regardless its contents.) environment, which respectively represent chemical and physical features of the air, are discretely and separately required for various aspects of development within the aspergilli. To more fully dissect the genetic processes involved in recognition of oxygen and the aeriform environment, we focused on the conidiogenesis process in *A. fumigatus* strain AF293, where we re-examined genetic mutants lacking regulators of this process in one, both or neither of the two stimuli. Detailed investigation revealed that conidiophore development is induced via an upstream Flbs and oxygen-dependent pathway in a gel-phase environment. Conversely, in an aeriform environment conidiophores are produced via an unknown pathway that stimulates the central regulator *AfubrlA* without oxygen and upstream Flbs. This evidence may explain differences between the model strains of *A. nidulans* and *A. fumigatus* (strain AF293), by suggesting a novel paradigm wherein aspergilli recognize “air” via two independent ways: one mediated by oxygen and a second responsive to the physical, aeriform state.

## Materials and Methods

### Fungal strains and growth conditions

Aspergillus strains used in this study are displayed in Table S1 (references cited in the table [22], [27], [28], [29], [30], [31], [32], [33], [34], [35], [36], [37], [38], [39], [40], [41], [42], [43], [44], [45]). All fungal strains were maintained on solid glucose minimal media (GMM, 1 % glucose), potato dextrose agar (PDA), and Sabouraud dextrose media (SAB) including 1.5 % (w/v) agar as previously described at 30 °C in the dark. Cellophane membrane (BioExpress, Kaysville, UT) was used for covering the surface of the GMM agar media to separate the fungal tissue into aerial and gel-embedded parts. To control aerial gaseous composition, fungal cultures were placed in sealable Ziploc® bags (S. C. Johnson & Son, Inc., Racine, WI) filled with ultra-high purity nitrogen or oxygen (James supplies & rental Co., Chickasaw, OK). Plates were placed in the incubator upside down to prevent condensation on the lid and accumulation of carbon dioxide, which may be produced from the fungal culture.

### Observation of conidiophore development

For each fungal strain used in this study, conidiophore development in the gel-phase environment was observed in PDA (or SAB) grown colonies covered with a cellophane membrane, which is permeable for oxygen, as described in detail in Figure S1..*A. fumigatus* colonies on GMM agar media were obtained by thin sectioning of 3–4 day old colonies, slide culture, or sandwiched culture (Figure S1). Note that spore inoculation sites for slide culture (Figure S1C) and sandwiched culture (Figure S1D) were the media-air interface and the middle of the agar block (gel-phase), respectively. Microscopy was done using Olympus BX41 and SZX12 microscopes equipped with an Olympus DP71 CCD camera (Olympus America Inc.).

### Isolation of RNA and quantitative reverse transcriptase PCR analysis

Sterile cellophane membranes (8 cm in diameter) were used to separate the aerial and the embedded tissues for gene expression analysis. Fungal tissues grown over the membrane were harvested by peeling the membrane off from the media, and those grown under the membrane were harvested by carving out the agar layer containing mycelia after peeling off the membrane. Total RNA was isolated from frozen fungal tissues with the RNeasy®plant mini kit (Qiagen, 74904) according to the manufacturer’s instruction. For quantitative RT-PCR, 5 µg of total RNA was reverse transcribed into first-strand cDNA with oligo (dT) primer using the SuperScript™ First-Strand Synthesis System (Invitrogen™ Life Technologies) according to the manufacturer’s instruction. Reactions were performed in a 10 µl volume containing 100 nM of each primer (see Table S3), 1 µg of cDNA and 5 µl of 2x Power SYBR® Green PCR Master Mix (Applied Biosystems, Warrington, UK). Quantitative PCR was run on the 384-well Applied Biosystems 7900 HT. After each run, amplification specificity was evaluated with a dissociation curve acquired by heating the samples from 60 to 95°C. Normalization and comparison of mean Ct values were performed as described [46]. To compare relative transcript abundance of target genes, the mean threshold cycle (Ct) of triplicate reactions was normalized to that of the β-tubulin gene of *A. fumigatus*. Quantitative RT-PCR was conducted at least three times with three technical replicates from independent biological experiments.

## Results

### Asexual development of *A. fumigatus* at the gel-embedded environment

**Figure 1:**
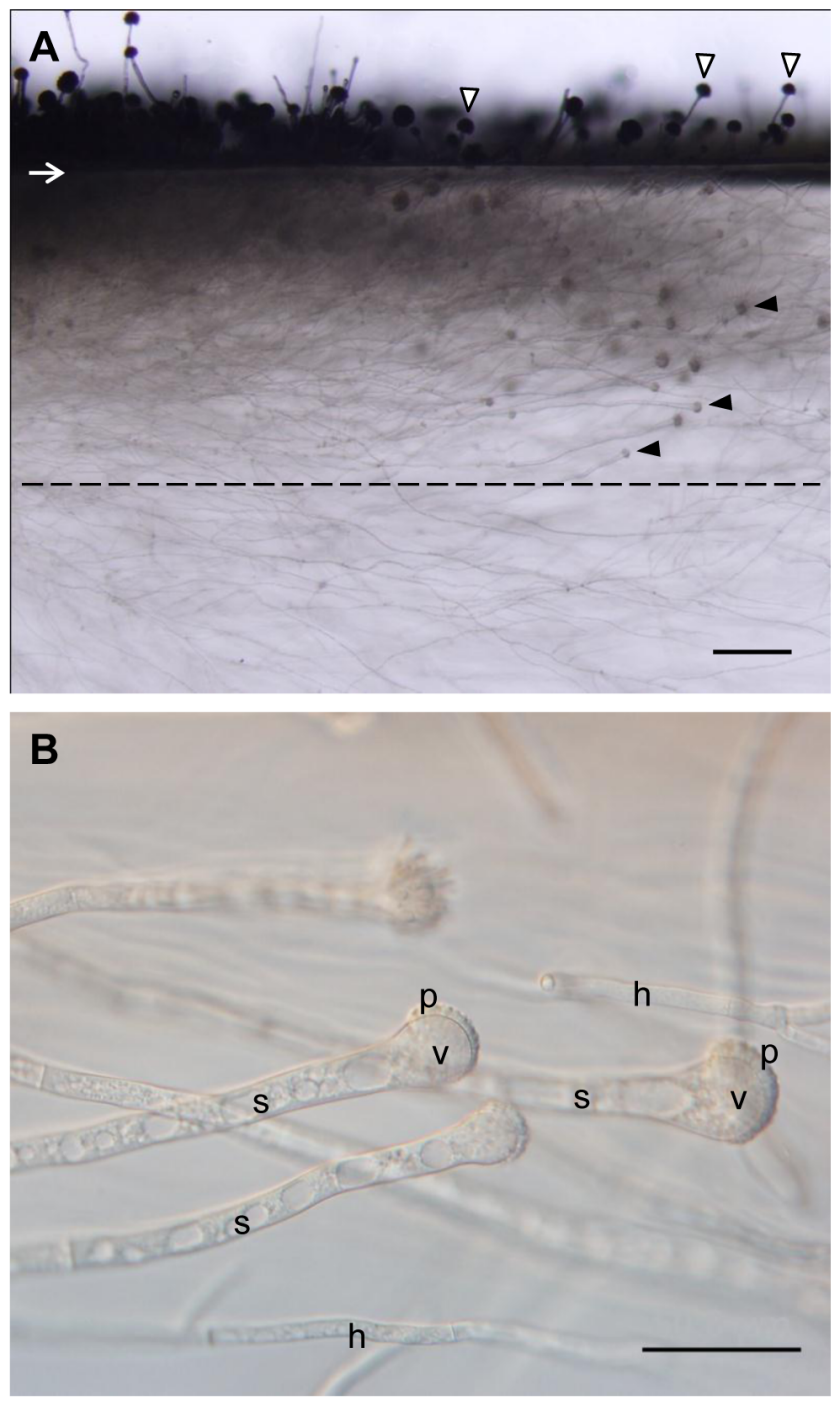
Development of *A. fumigatus* strain AF293 in aerial and gel-embedded environments. Vertical projection of *A. fumigatus* AF293 colony is shown. Conidial suspension was inoculated and cultured on a thin section of GMM agar (4×30×10 mm) using the slide culture method (see Figure S2 for detailed method). Pictures were taken at 72 hours after inoculation. (A) Overview at low magnification. The margin between the agar and the air is indicated with a white arrow. Some aerial conidiophores are indicated by white arrowheads, and gel-phase conidiophores are indicated by black arrowheads. Note that no conidiophores were found in the deeper area (under the dashed line). Bar  =  200 µm. (B) Conidiophores in gel-phase environment. Vacuolated stalks (s), vesicles (v), phialides (p), and invasive hyphae (h) are indicated. Bar  =  50 µm.

**Figure 2:**
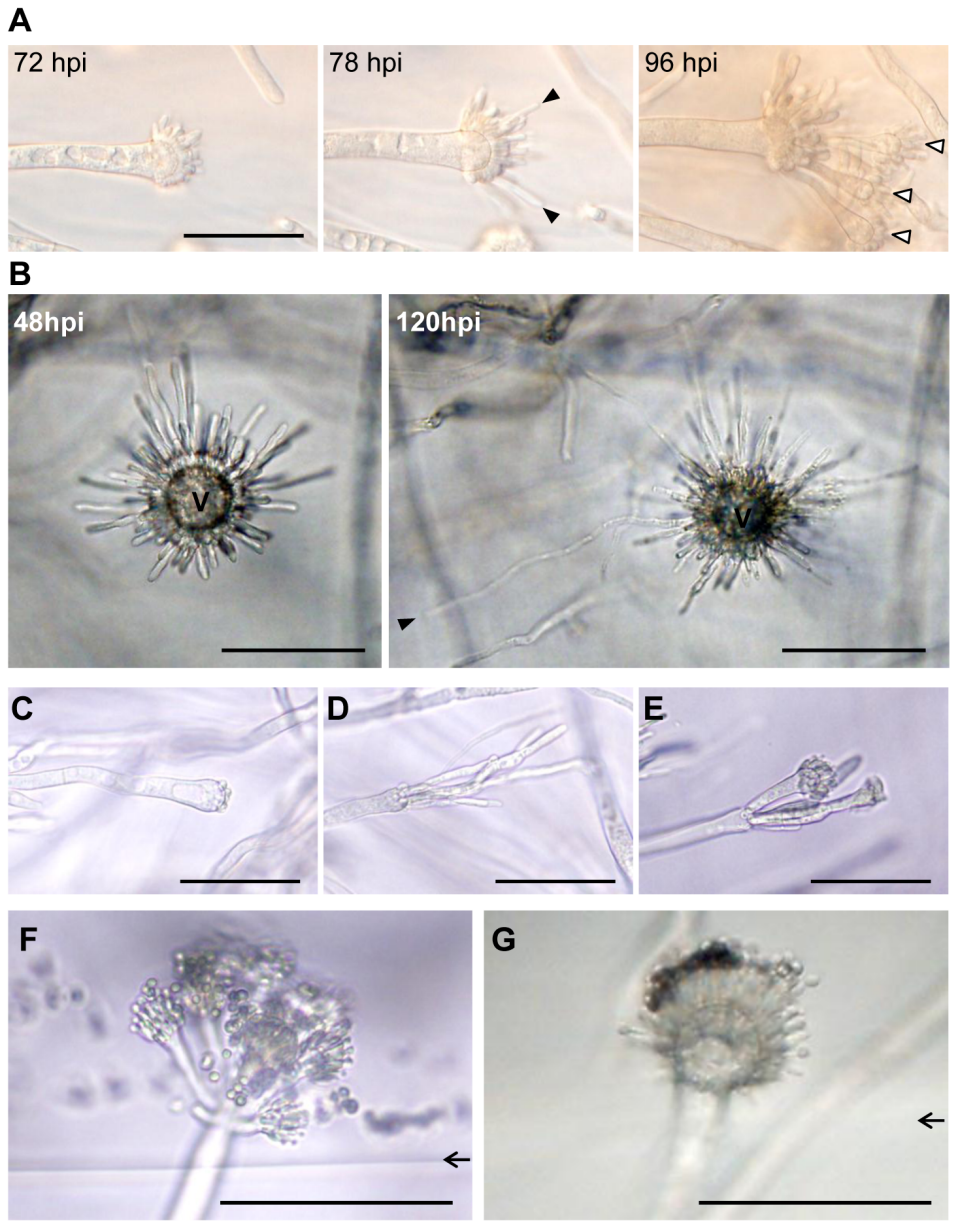
Characteristics of gel-phase conidiophores in *A. fumigatus.* Pictures of gel-phase conidiophores were taken from slide culture (see Figure S2) of strain AF293 embedded in GMM agar media from 48 to 120 hour after inoculation. Bars  =  50 µm. (A) Time lapse tracking of a conidiophore developing secondary conidiophores. Elongated phialides (black arrowheads) and secondary conidiophores (white arrowheads) were observed at 78 hour and 96 hour after inoculation, respectively. (B) Time lapse tracking of embedded phialides associated with a vesicle (v). An extensively elongated phialide than the others (at 120 hour) is marked with black arrowheads. (C-E) Various morphologies of conidiophores in the gel-phase environment. (F-G) Conidiophores in transition stage between gel and air (basal stalks are embedded; but vesicles, phialides and conidia are exposed to air). Arrows indicates boundary interface between aerial and agar layer.

We discovered developing conidiophores of *A. fumigatus* (strain AF293) in a gel (glucose minimal agar media, solid)-embedded substrate (Figure 1). Morphologically, the embedded structures appeared similar to stalks, vesicles and phialides of conidiophores and are clearly distinguishable from vegetative hyphae (Figure 1B). Gel-embedded conidiophores were only observed in the upper agar layer but not in deeper layers, where branched, invasive hyphae predominated (∼600 µm below the agar-air interface, dashed line in Figure 1A). The gel-embedded stalks were usually highly vacuolated (Figure 1B), and differentiated from thick, subtending hyphae from which they were separated by a septum. Subtending hyphae undergoing stalk development remained unbranched and their position and orientation were primarily towards the colony periphery and the aerial surface, respectively (Figure 1). Despite these similarities, gel-embedded conidiophores have distinct characteristics from aerial ones. The most significant difference between gel-embedded conidiophores and their aerial counterparts was that conidiophores developing in the subsurface completely lacked conidia (see below). Time lapse tracking of embedded, developing conidiophores revealed that instead of giving rise to conidia, phialides either elongated apically or differentiated into secondary conidiophores (Figure 2A and 2B, respectively). Like the gel-embedded stalks, the elongating phialides were not branched, and their apical extension was very slow (e.g. the phialide in Figure 2B, which is marked with black arrowhead, extended ∼70 µm in 72 hours) compared to that of vegetative hyphae. The morphology of embedded conidiophores varied in terms of thickness of stalks, vesicle size, and number of phialides (Figure 2C, 2D and 2E). Further, the length and size of elongated phialides was variable, and the developmental progression into secondary vesicles was not synchronized even among phialides from the same primary vesicle (Figure 2A). Despite this seemingly abnormal development, conidia production was restored once the gel-embedded conidiophore reached the air surface (Figure 2F and 2G). Conidia were observed not only on solitary stalks emerging from the gel (Figure 2G) but also on secondary conidiophores differentiated from elongated phialides (Figure 2F). Intriguingly, conidial differentiation and pigmentation was observed to occur on phialides located on the top of the emerging vesicle (Figure 2G), suggesting that air-contact (or release from gel) is required for each phialide cell to produce conidia. From these results, we conclude that stalks, vesicles and phialides of *A. fumigatus* can differentiate within the physical matrix of the agar, while the development of conidia from phialides is seemingly inhibited.

### The role of oxygen and physical transition to an ‘aeriform’ environment as signals for conidiogenesis in *A. fumigatus* AF293

**Figure 3:**
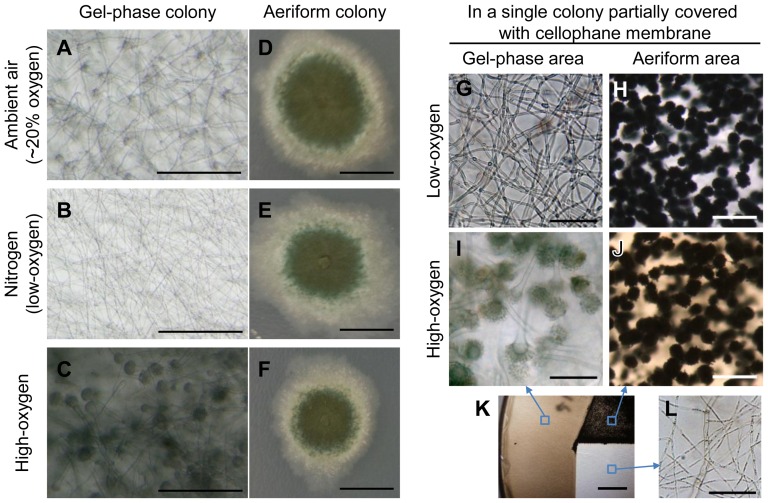
Effect of oxygen and aeriform in conidiogenesis of *A. fumigatus* strain AF293. Left panels: AF293 colonies in gel-phase or aeriform under ambient air (A and D), nitrogen (low oxygen, B and E) or high-oxygen (C and F). Pictures were taken 96 hours after point inoculation. (A-C) Colonies under gel-phase environment. Bars  =  200 µm. (D-F) Colonies in aeriform environment. Colony areas in pale color are made up with creeping and aerial hyphae, and areas in green color represent conidiophores with mature conidia. Bars  =  1 cm. Right panels: Single AF293 colonies partially covered with cellophane membrane, which was cultured in low oxygen condition for 6 days (G and H) or cultured in low-oxyen condition for 4 days then transferred and cultured in high-oxygen condition for 2 days (I - L). (G and I) Pictures from cellophane covered area. Bars  =  50 µm. (H and J) Pictures from the uncovered (aeriform) area. Bars  =  200 µm. K. AF293 colony partially covered with cellophane membrane (left), glass coverslip (right bottom), and nothing (right top). Blue squares indicate the location of magnified pictures (I, J and L) taken. Bar  =  5 mm. (see Figure S2A for illustrated method.) L. AF293 colony covered with glass coverslip in high-oxygen condition. Bars  =  50 µm.

**Figure 4:**
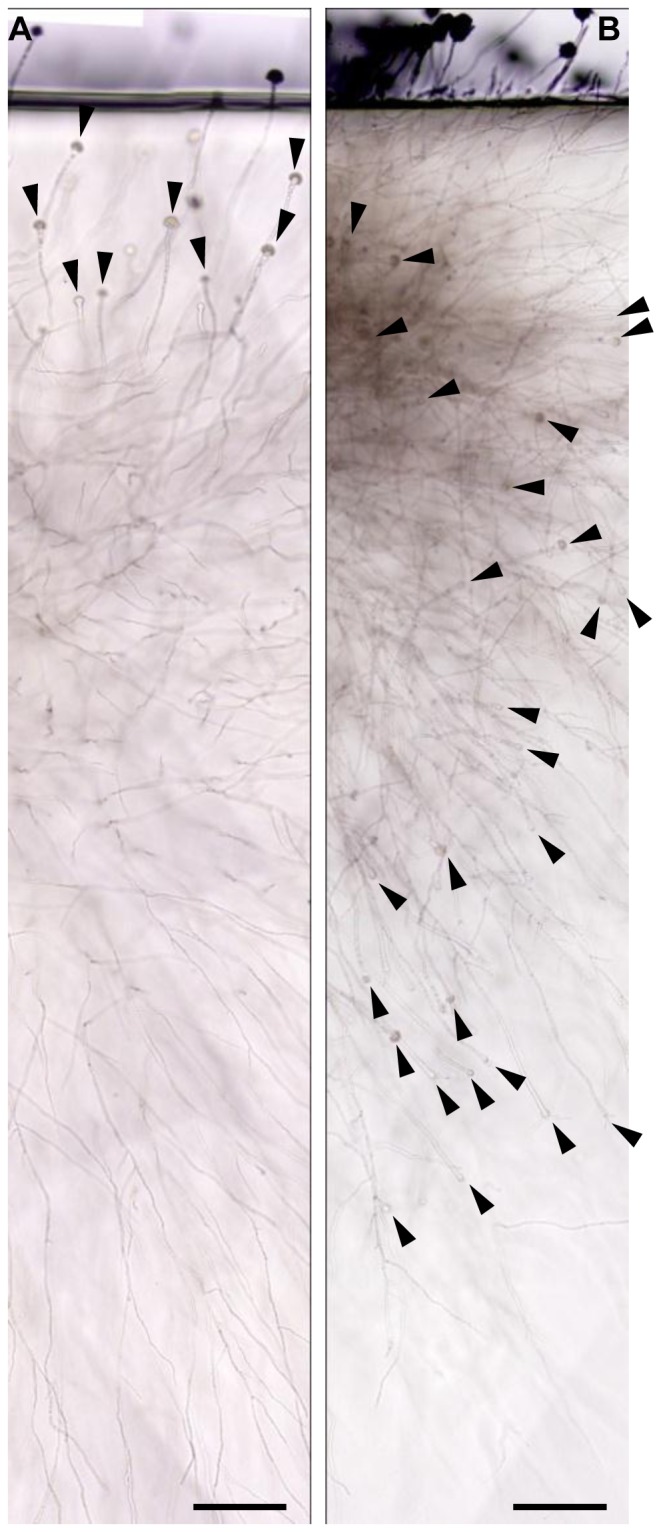
Oxygen dependent development of *A. fumigatus* conidiophore. *A. fumigatus* (AF293) colonies grown on thin agar blocks of GMM using the sandwiched culture method (see Methods and Figure S2). Series of pictures were concatenated by using photomerge function of Adobe Photoshop Elements 9. Pictures were taken 72 hours after inoculation. The locations of embedded vesicles are indicated by black arrowheads. Bars  = 100 µm. (A) Colonies in ambient air. (B) Colonies in oxygen-saturated agar block.

Since the conidiophores in the gel environment were only found near the surface, while invasive hyphae were found in deeper layers, and the orientation of the former was mainly towards the agar surface (Figure 1A), we hypothesized that soluble or permeable air components (such as oxygen) in the agar might be required for developmental reprogramming to conidiogenesis. To evaluate this possibility, we compared the propensity for conidiogenesis in normal (ambient air), a highly oxygenated condition, and a hypoxic condition. Intriguingly, conidiophore development in the gel-environment was dramatically influenced by the oxygen level. In the hypoxic (low-oxygen) condition, no stalks or vesicles were observed in the gel-embedded colony, while many conidiophores were commonly observed in the ambient air condition (∼20% oxygen) and high oxygen condition (Figure 3A-C). Furthermore, the density of conidiophores and the level of pigmentation in the vesicles were much higher in the high-oxygen condition than in ambient air (Figure 3A and 3C). This suggests that the initiation of conidiogenesis in the agar matrix is dependent upon oxygen concentration in *A. fumigatus* AF293. In addition, analysis of a vertical projection of AF293 colonies revealed that gel-embedded conidiophores, normally confined to the agar-air interface and oriented towards the agar surface (Figure 4A) developed throughout the colony, with stalk elongation progressing in all directions towards the colony periphery, irrespective of their position relative to the surface when the agar block was saturated with oxygen (Figure 4B). This further implicates oxygen as not only an essential factor for initiation of conidiogenesis, but as a potential compass for determining directionality of conidiophore development in the gel-embedded environment.

In stark contrast, the oxygen dependency implicated in the subsurface environment was not observed in the ‘aeriform’ environment. Colonies grown on top of the cellophane membrane produced mature conidiophores with green-pigmented conidia regardless of the oxygen level, although the growth rate of the colonies varied slightly (Figure 3D-F). To confirm these developmental shifts occur specifically in response to oxygen and/or an ‘aeriform’ environment, colonies of AF293 were cultured under cellophane membrane with an opening to expose the mycelia in a single colony to the gel-embedded and ‘aeriform’ environment simultaneously (Figure 3K and S2A). To obtain developmental competency and synchronized growth, the colonies were cultured in a low-oxygen condition for 4 days and then transferred to a high-oxygen condition or kept in the low-oxygen condition, to induce or suppress development, respectively. In the oxygen-induced condition, lots of conidiophores were observed both in the cellophane-covered area and a region where the cellophane had been cut out (Figure 3I and 3J). Without oxygen induction, no conidiophores were found under the cellophane membrane as previously observed (Figure 3G), however, lots of conidiophores were found in the cut away region (Figure 3H). These results indicate that the transition from a gel-embedded environment to an aeriform one is a sufficient cue for triggering conidiophore development, regardless of the oxygen concentration. Furthermore, even in the oxygen induced condition, no conidiophores were found in an area covered with an oxygen-impermeable glass coverslip (Figure 3L), indicating that the oxygen-mediated induction occurs locally in mycelia directly contacting oxygen (i.e. directly under the cellophane membrane). Pigment production also seems to be linked to oxygen but not necessarily with conidiophore development because orange coloration was detected only in oxygen exposed areas (Figure 3J and 3K) and not in low-oxygen/aeriform area where lots of conidiophores were produced (Figure 3H).

In summation, these results suggest that conidiophore development of *A. fumigatus* (strain AF293) is induced by either of two distinct features of air; one is oxygen and the other is the phase transition to the aeriform environment itself.

### Developmental response to oxygen and the aeriform environment varies among aspergilli

To determine whether asexual development in response to oxygen and the physical environment is common among the aspergilli, conidiophore development of 19 additional strains (contained within 16 species) in this genus were observed in high and low oxygen conditions in both aeriform and gel-embedded environments (Table 1 and Figure S2). Sixteen strains produced typical conidiophores in at least one of the given conditions while three of them (type E; Figure S1) didn’t produce conidiophores (or similar structures) in any condition. A third, *Dichotomomyces cejpii* (type È) produced atypical conidiophores in all conditions tested (Table 1 and Figure S2). Thus, while all of the tested strains (except type E) altered their development in response to oxygen and/or physical environment, most did so in unique ways with one or the other serving as the dominant stimulus. Based on these developmental distinctions, we categorized them into four major types (Table 1). Four strains belonging to type A showed similar developmental behavior with AF293 wherein aeriform development was oxygen-independent and gel-phase development was oxygen-dependent. However, the size of the conidiophore heads (quantitative expansion of conidial chains) of two strains (*A. kanagawaensis* strain NRRL5023 and *A. nidulans* strain FGSC A117) was still dependent on oxygen (Figure S2). This suggests that while either oxygen or ’aeriform’ is sufficient for initiation of conidiophore development in these two strains, both of them are required for maximal conidial development. One strain belonging to type B (*A. unilateralis* NRRL577) developed conidiophores only in response to oxygen regardless of the physical environment (Table 1, and Figure S2). However, the gel-phase conidiophores of this strain terminated differentiation at the phialide stage, similarly to AF293 (and type A strains, Figure S2). This indicates that, in these strains, initiation of asexual development can be induced by oxygen alone but an aeriform environment is still required for terminal differentiation of conidia. In contrast, two strains (*A. niger* FGSC A732 and *A. nidulans* FGSC A90) grouped in type C were able to produce conidiophores only in an aeriform environment, regardless of oxygen, but their size and number were still affected by oxygen concentration (Figure S2). Lastly, the majority of strains (9 of 20), including the model strain of *A. nidulans* (FGSC A4), were classified into type D, and require both oxygen and an aeriform environment for conidiophore development. Based on these results, we propose that oxygen and the aeriform condition may be required for complete asexual development of most aspergilli. In addition, the majority of aspergilli tested in this study showed other developmental changes in response to oxygen and/or the gel-phase environment (e.g. increase in pigment production, see Figure S3), even in strains belonging to types C, D and E, which didn’t produce conidiophores in the gel-phase environment. This indicates that, even in a gel-embedded environment, oxygen still can induce significant developmental changes (besides conidiogenesis) in most aspergilli.

**Table 1:** Conidiophore development of various aspergilli in response of oxygen and aeriform environment.^a)^

Species name	Strain ID	Aeriform		Gel-phase		Type	Section	Note
		High-oxygen	Low-oxygen	High-oxygen	Low-oxygen			
*Aseprgillus fumigatus*	AF293	O^b)^	O	O	X^C)^	A	*Fumigati*	FGSC A1100
*Aspergillus parvulus*	NRRL 2667	O	O	O	X	A	*Cervini*	
*Aspergillus kanagawaensis*	NRRL 5023	O	O*	O	X	A	*Cervini*	*reduced in size
*Aspergillus nidulans*	FGSC A117	O	O*	O	X	A	*Nidulantes*	*reduced in size
*Aspergillus unilateralis*	NRRL 577	O	X	O	X	B	*Fumigati*	
*Aspergillus niger*	FGSC A732	O	O*	X	X	C	*Nigri*	*reduced in size and number
*Aspergillus nidulans*	FGSC A90	O	O*	X	X	C	*Nidulantes*	*reduced in size and number
*Aspergillus awamori*	FGSC A808	O	X	X	X	D	*Nigri*	
*Aspergillus terreus*	FGSC A1156	O	X	X	X	D	*Terrei*	
*Aspergillus terreus*	NRRL 260	O	X	X	X	D	*Terrei*	
*Aspergillus oryzae*	FGSC A815	O	X	X	X	D	*Flavi*	
*Aspergillus nidulans*	FGSC A4	O	X	X	X	D	*Nidulantes*	
*Aspergillus giganteus*	NRRL 10	O	X	X	X	D	*Clavati*	
*Aspergillus clavatus*	NRRL 1	O*	X	X	X	D	*Clavati*	*not abundant
*Neosartorya pseudofischeri*	NRRL 20748	O*	X	X	X	D	*Fumigati*	*not abundant
*Aspergillus heterothallicus*	FGSC A251	O	X*	X	X	D'	*Usti*	*growth defective
*Neocarpenteles acanthosporum*	NRRL5293	X	X	X	X	E	*Clavati*	
*Neosartorya aureola*	NRRL2244	X	X	X	X	E	*Fumigati*	
*Neosatorya fischeri*	NRRL181	X	X	X	X	E	*Fumigati*	
*Dichotomomyces cejpii*	NRRL 26980	O*	O*	O*	O*	E'	*Clavati*	*not typical conidiophore

a) Agar blocks containing active growing mycelia of the twenty aspergilli were inoculated on PDA and cultured in high or low oxygen condition with or without cellophane membrane cover on the colony. Conidiophore development was determined at 60 hours after inoculation. Pictorial replication of this table is shown in Figure S1.

b) O  =  Conidiophores were found in the given condition.

c) X  =  No conidiophore was found in the given condition.

Interestingly, classification of the strains herein by their developmental behavior (type A to E) is not correlated to phylogenetic affinity (Table 1). For example, the developmental behaviors of three *A. nidulans* strains (FGSC A4, A90 and A117) were clearly distinct from each other (Table 1, and Figure S2). Further, we investigated fourteen additional *A. fumigatus* strains in the gel-embedded environment to determine whether their developmental behavior could be correlated to any of a number of other traits. Eight isolates of *A. fumigatus* produced the gel-embedded conidiophores while the other six isolates didn’t (Table S2). Among those grouping together in this developmental behavior, we could find no correlation to other characteristics including mating type, geographical location or whether the strains were isolated from a clinical or environmental setting (Table S2).

### The oxygen-dependent initiation of conidiogenesis in *A. fumigatus* AF293 requires known upstream regulators

**Figure 5:**
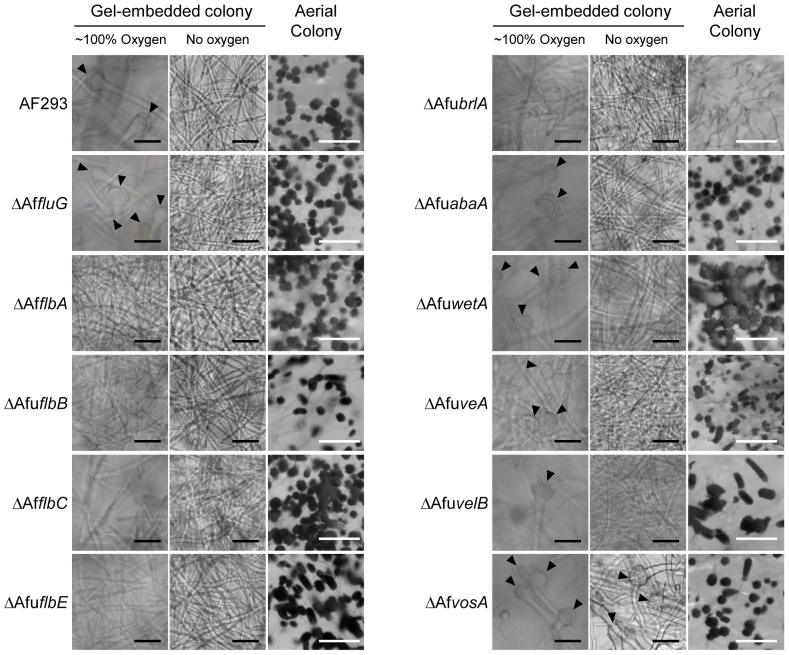
Conidiophore development of the mutants lacking regulatory genes under gel-phase and aerial environments. *A.fumigatus* mutants and WT (AF293) strains grown in gel-phase with or without oxygen (left and middle panel, respectively, bars  =  30 µm) and under aeriform environment (right panel, bars  =  200 µm). Pictures were taken 96 hours after inoculation. The locations of embedded vesicles are indicated by black arrowheads.

To further explore the relationship between the environmental factors identified in this study and known regulatory genes, *A. fumigatus* AF293 was selected as a model system since many mutants lacking genes controlling various regulatory checkpoints throughout the conidiogenesis pathway are already available. We investigated the developmental phenotype of mutants lacking upstream regulators (*AfufluG*, *AfuflbA*, *AfuflbB*, *AfuflbC*, and *AfuflbE*), central regulators (*AfubrlA*, *AfuabaA*, and *AfuwetA*) and downstream regulators (*AfuveA*, *AfuvelB*, and *AfuvosA*) in the gel-phase environment with or without oxygen (see references for these mutants in Table S2). As we observed previously, the wild type AF293 produced conidiophores in high-oxygen conditions but not in low-oxygen conditions in the gel-phase environment (Figure 5). The mutant lacking *AfufluG*, known as one of the most upstream components [16], [27], showed the same phenotype as AF293 in the given conditions (Figure 5), indicating *AfufluG* is dispensable for oxygen-mediated conidiogenesis. Intriguingly, the mutants lacking the upstream regulators (*AfuflbA*, *AfuflbB*, *AfuflbC*, and *AfuflbE*) didn’t produce any gel-phase conidiophores in high-oxygen conditions up to 96 hours after inoculation, while readily developing aerial conidiophores in the aeriform condition (Figure 5). This suggests that the upstream regulators (Flbs) are required for oxygen-mediated conidiogenesis in the gel-embedded environment but not for aeriform-mediated development. The mutants lacking the negative regulator, *AfuvosA*, produced gel-phase conidiophores even without oxygen while mutants lacking the other *velvet* regulators (*AfuveA* and *AfuvelB*) produced gel-phase conidiophores only in high-oxygen conditions (Figure 5). This suggests that suppression of *AfuvosA* is sufficient for conidiophore development without induction from oxygen or an aeriform environment. The mutant lacking a key transcriptional regulator, *AfubrlA*, didn’t produce conidiophores in any condition tested, but the mutants lacking *AfuabaA* and *AfuwetA* produced both gel-phase and aeriform conidiophores although the phenotype of aeriform ones was altered from the wild type (Figure 5, see reference [28] for detailed phenotypes). Since the phenotype of the gel-phase conidiophores of AF293 were indistinguishable from that of the *▵AfuabaA* mutant (elongated phialides and no conidial chains), we hypothesized that the inhibition of conidia production observed in gel-phase conidiophores might be due to suppression of *AfuabaA* expression (see below).

### Oxygen induced gel-phase conidiophore development involves up-regulation of *AfubrlA* and down-regulation of *AfuabaA*

**Figure 6:**
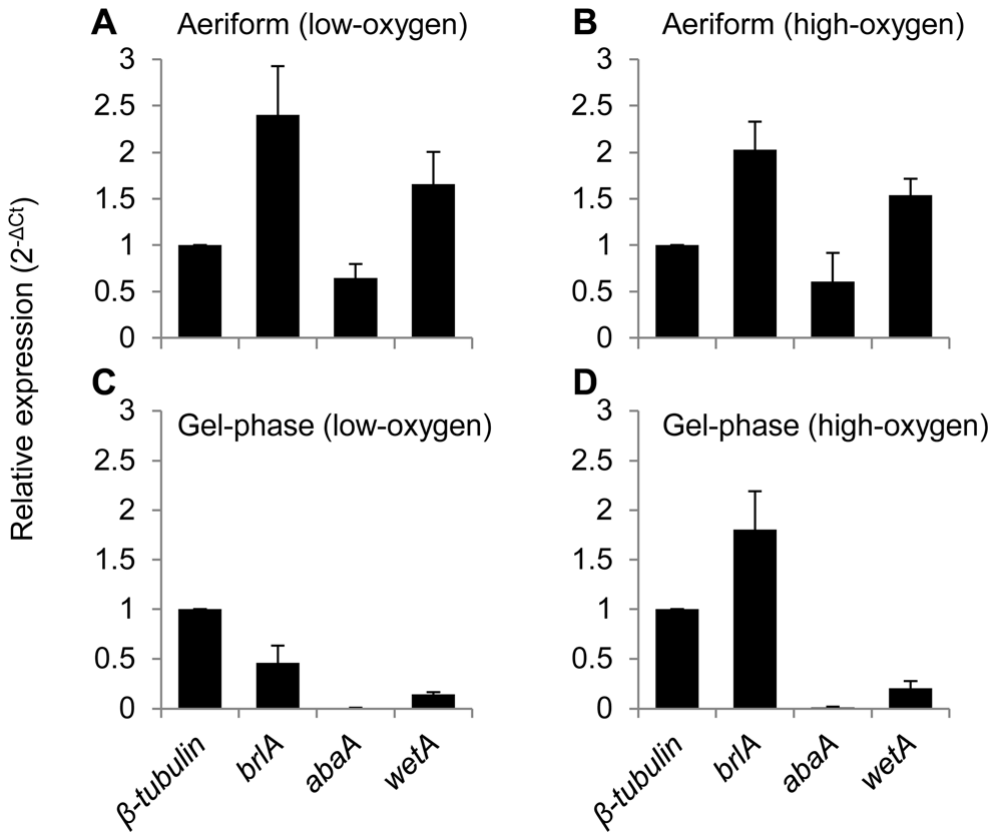
Expression of central regulatory genes of *A. fumigatus* in response to oxygen and physical environments. Total RNA extracted from *A. fumigatus* colonies grown in gel-phase or aeriform environment with or without oxygen (see figure 5). Colonies were cultured on GMM agar media from upside (aeriform colony) or downside (gel-phase colony) of a cellophane membrane for 96 hours at 30°C. Bars are mean values and error bars are standard deviation of relative expression levels normalized with Ct values of β-tubulin. n  =  3. Environmental conditions for the expression analysis are as follows; (A) aeriform and low-oxygen condition, (B) aeriform and high-oxygen condition, (C) gel-phase and low-oxygen condition, and (D) gel-phase and high-oxygen condition.

To further elucidate the genetic mechanism(s) involved in gel-phase conidiophore development in AF293, we examined the expression level of genes governing distinct stages of conidiogenesis (*AfubrlA*, *AfuabaA*, and *AfuwetA*) [27], [28]. We extracted RNA from aeriform and gel-phase colonies grown under high- and low-oxygen conditions at 96 hours (as shown in Figure 3), and the expression levels of *AfubrlA*, *AfuabaA*, and *AfuwetA* were compared using quantitative RT-PCR. The expression level of *AfubrlA*, the key regulatory factor promoting early conidiophore development [27], was highly expressed (more than twice of β-tubulin level) in the aeriform environment regardless of the presence of oxygen (Figure 6A and 6B). Since aeriform signaling doesn’t seem to require the upstream regulators (Flbs in Figure 5), it is presumed that AF293 might have an independent pathway from Flb to induce the expression of *AfubrlA*. In contrast, *AfubrlA* expression was highly dependent upon the presence of oxygen in the gel-phase environment, being down-regulated (less than half of β-tubulin level) in the absence of oxygen and up-regulated (∼1.8 times of β-tubulin level) in the presence of oxygen (Figure 6C and 6D). This is congruent with our phenotypic observations in Figure 3, and indicates that either one of the aeriform environment and oxygen is sufficient for up-regulation of *AfubrlA* expression and subsequent conidiophore initiation.

In contrast to *AfubrlA*, a distinct expression behavior was observed in *AfuabaA*, known as a direct downstream component of *AfubrlA* in the conidiogenesis transcriptional pathway and a key regulator for differentiation of phialides into conidia [28], [47], [48]. The expression level of *AfuabaA* in aeriform environments was quite high (∼60% of β-tubulin level, Figure 6A and 6B), but expression was almost undetectable (∼ 1% of β-tubulin level) in the gel-phase environment (Figure 6C and 6D), regardless of the oxygen level. In particular, *AfuabaA* expression remained at low levels even when the direct upstream regulator, *AfubrlA*, was highly up-regulated by oxygen (Figure 6D). This indicates that the gel-phase environment represses the expression of *AfuabaA*, regardless of *AfubrlA* activity. Intriguingly, the phenotype of the *AfuabaA* mutant reported previously [28] is very similar to what we observe with the wild type conidiophores grown in the gel-phase environment (Figure 2), with phialides apically elongating yet failing to produce conidia. This strongly suggests that the repression of terminal conidiogenesis stages we observe in the gel-phase environment results from a lack of *AfuabaA* gene expression. Likewise, the regulatory gene *AfuwetA,* which acts downstream of *AfuabaA* and is responsible for maturation of conidia [12], [28], was expressed at relatively low levels in the gel-phase environment when compared to those in the aeriform environment (Figure 6).

## Discussion

### Environmental factors involved in conidiogenesis of aspergilli

The sensing of environmental conditions is critical for filamentous fungi to successfully produce and disseminate their reproductive propagules, or conidia. Thus, investigation of the relationship between environmental factors and the subsequent intracellular regulatory mechanisms in fungi is required for a better understanding of these developmental processes. Among the aspergilli, the emergence of hyphae into an aerial environment is considered a major cue for conidiogenesis [1], [5] because this development is generally not induced in submerged environments (e.g. in liquid media) without nutrient starvation [49] or salt stress [50]. However, very little is known regarding how fungi recognize the aerial environment thus far. In this study, we report that both oxygen and a phase transition in the extracellular environment can independently regulate conidiogenesis in many aspergilli. Oxygen was essential for asexual development of the most aspergilli tested in this study; in low-oxygen conditions, 10 strains (out of 16) were not able to produce any conidiophores (type B and D, Table 1), and 4 strains produced conidiophores of reduced size and/or quantity (part of type A and type C, Table 1). Although two strains (*A. fumigatus* AF293 and *A. parvulus* NRRL 2667) produced normal conidiophores in aeriform conditions without oxygen, they still required oxygen in the embedded, gel-phase environment (Table 1). Furthermore, oxygen appeared to direct the orientation of conidiophores (Figure 4), and the response to oxygen was dosage dependent in terms of conidiophore quantity and pigmentation (Figure 3), and it occurred locally only in oxygen-exposed areas (Figure 3, I-L). From this result, we suggest that oxygen is a prerequisite for asexual development in most aspergilli. However, pinpointing oxygen as the environmental cue is premature, because a sensing mechanism directing the regulatory pathway is thus far unknown. Further, it is possible that the oxygen effects we have observed might be a result of oxygen-induced physiological changes, such as an increase in respiration and the production of TCA cycle intermediates, which stimulate vesicle and phialide formation in *A. niger* [51], [52], and reactive oxygen that stimulates various aspects of fungal development [53]. Yet, considering the role of oxygen for asexual development strictly from a metabolism standpoint doesn’t seem appropriate either, at least in strains belonging to type A and C, since these fungi develop conidiophores without any oxygen supply (Table 1 and Figure S2).

Intriguingly, this study also identified the physical aeriform environment as a cue for proper asexual development in most aspergilli tested. In fact, four strains (type A; Table 1) were able to produce conidiophores in an aeriform environment without oxygen. Since the development of these strains was still dependent on oxygen in the gel-phase environment, it is assumed that being in an aeriform environment is indeed a cue for conidiogenesis in these strains. Furthermore, gel-phase conidiophores in these strains were defective in conidial chain differentiation (Figure 2 and S2) indicating conidiogenesis is still suppressed in the physically embedded environment. This is likely due to genetic repression in response to the physical environment rather than physical pressure itself because transcriptional repression of *AfuabaA* and differentiation of elongated phialides and secondary conidiophores were observed in the gel-phase environment (Figure 2 and 6). Additional evidence supporting the aeriform condition as a prerequisite for conidiophore development is that eleven strains (type C and D) didn’t produce any conidiophores in the gel-phase environment, even in high-oxygen conditions. This is not likely due to insufficient oxygen supply or availability because most of these strains produced higher levels of pigment in response to oxygen in this same gel-embedded area. However, we couldn’t rule out this possibility since the oxygen requirement of each strain and precise concentration of oxygen in the gel-phase environment are still unknown. Overall, we suggest that most aspergilli require oxygen and an aeriform environment as signals for recognizing air and developing aerial conidia, although their respective requirements (or dominance over each other) vary depending upon the species and strains.

### Role of upstream and downstream regulators in “air recognition” signaling in aspergilli

The differences we observed in conidiogenesis behavior between aspergilli may be due to distinctions in an environmental sensory mechanism. These same distinctions may also explain the phenotypic gap between the loss-of-function mutants lacking *flbs* in *A. fumigatus* and *A. nidulans* [22], [27], [30]. In *A. nidulans*, FluG is known as the most upstream regulator of conidiogenesis, producing an extracellular sporulation-inducing factor [16], [54], and FluG-mediated development is processed via upstream Flbs [55]. However, AfuFluG in strain AF293 seemed to not only be dispensable for aerial conidiogenesis (Figure 5, [27]) but also to partially contribute to gel-phase conidiogenesis because the *&utri;AfufluG* mutant produced normal conidiophores in the gel environment (Figure 5), although the developmental timing was somewhat delayed in a liquid environment [27]. Furthermore, *A. fumigatus* (AF293) mutants lacking genes in the upstream regulatory pathway (Flbs) were completely defective in oxygen-induced development in the gel-phase environment while they were able to form conidiophores in the aeriform condition (Figure 5). *A. nidulans* mutants lacking the same Flbs were defective in conidiogenesis in the aeriform condition where the *A. fumigatus* mutants were not [18], [19], [20], [21], [22]. Since oxygen is imperative for conidiophore development of *A. nidulans* (even in aeriform) and *A. fumigatus* when it is embedded in gel-phase (note that it could induce the development in aeriform without oxygen), these results lead us to suggest that oxygen-dependent conidiogenesis requires the upstream Flbs and *A. fumigatus* might have an additional regulator which turns on *AfubrlA* expression and conidiogenesis without the oxygen dependent, upstream Flbs. AfuFlbA (as well as FlbA in *A. nidulans*) is an RGS (regulator of G protein signaling) targeting GpaA (Gα) and stimulating *AfubrlA* expression via negative regulation of GpaA-mediated signaling for vegetative growth [17], [18], [27]. The AfuFlbB is predicted to be a potential TF (transcription factor) containing a b-zip DNA binding domain, and it functions with AfuFlbE (containing unknown domain) inter-dependently for expression of AfuFlbD (containing a c-Myb DNA binding domain) and finally for proper expression of *AfubrlA* and conidiogenesis [22], [30]. The molecular functions and regulatory mechanisms of upstream Flbs in *A. fumigatus* are almost identical to the homologs in *A. nidulans*, and some of them are even cross-complementable between the two species [22], [30]. Therefore, the difference between mutants lacking Flbs in *A. nidulans* and *A. fumigatus* (AF293) might be rather due to a distinction in an environmental sensory system than functional differences in upstream Flbs. It is expected that there is a suppression mechanism of conidiophore development in the gel-phase not only because type D strains were not able to produce conidiophores in the gel-phase environment where sufficient oxygen was supplied for induction of other development including pigment production, but also because type A and B strains were not able to differentiate conidial chains from their gel-embedded conidiophores (Figure S2). Expression analysis led us to find that the gel-phase repression in *A. fumigatus* AF293occurs by transcriptional repression of *AfuAbaA* even though *AfuBrlA* was highly induced by oxygen (Figure 2 and 6). Our working hypothesis explaining the difference between *A. nidulans* A4 (type D) and *A. fumigatus* AF293 (type A) is that there is a suppression mechanism active in the gel-phase environment, likely to be genetic element(s) controlling transcription of regulatory genes, suppress upstream components (of Flbs) such as SfgA or FluG in the former and suppress AbaA downstream component in the latter.

We also found that the phenotype of *velvet* regulators in the gel-phase environment was distinct from their phenotype in liquid environments observed in previous studies. Park et al (2012) recently suggested that velvet regulators negatively regulate the expression of *AfubrlA* and conidiogenesis of *A. fumigatus*, since the various mutants lacking the *velvet* regulators, AfuVosA, AfuVeA and AfuVelB, produced conidiophores, and *&utri;AfuveA* and *&utri;AfuvelB* even produced green conidia in the liquid environment at 24 hours, while the wild type remains in a hyphal stage [24]. Adding to this phenomenon, we found that the developmental de-repression of *&utri;AfuveA and &utri;AfuvelB* did not occur in the gel-phase environment without oxygen, even after 96 hours (Figure 5), which indicates that the acceleration of conidiogenesis through lack of *AfuVeA* or *AfuVelB* is still dependent on oxygen. In contrast, conidiophore development in a hypoxic gel-phase environment was observed only in *&utri;AfuvosA* (Figure 5), suggesting that AfuVosA might directly regulate *AfubrlA* and the initiation of conidiogenesis regardless of oxygen. Collectively, we identified novel aspects of upstream and downstream components controlling conidiogenesis in *A. fumigatus*, which provides advanced understanding of the regulatory mechanism(s) involved.

### Gel-phase development in *A. fumigatus*: a useful feature for studying the conidiogenesis process in detail

Finding a well-defined artificial condition that induces particular development of an organism is very important not only because it reveals the detailed relationship between environmental factors and the development they stimulate, but also because it facilitates other studies in developmental biology. For example, gel-phase conidiogenesis in cellophane covered colonies provides an advanced platform to study conidiogenesis of *Aspergillus fumigatus* AF293 because the development is easily turned on and off by controlling the oxygen concentration and it provides a spore-free experimental environment, allowing direct observation of the asexual development process, including the major morphological differentiations (stalks, vesicles, phialides, and pigments).

Furthermore, conidiophores in an agar matrix enable cell imaging and various staining techniques. Previously, highly pigmented and hydrophobic conidia have hindered light transmission and staining with water-soluble dyes. The gel-embedded condition also permits live cell tracking for a more comprehensive observation of developing conidiophores up to 120 hours (e.g. by using fluorescently labeled proteins). We observed that neither phialides nor stalks resume normal vegetative growth with branching even after extended periods in culture (Figure 2B). Further, phialides released from the embedded environment immediately differentiate into conidia-bearing structures (Figure 2F and 2G). This might imply that while cellular modules for conidiogenesis are developmentally flexible among stalks, vesicles and phialides, they are not totipotent and once conidiogenesis is initiated, resumption of vegetative growth is prevented in those cells. Besides, the reduction in size noted in secondary conidiophores differentiated from elongated phialides (Figure 2A) may be linked to a lack of nutrient acquisition ability and limited supply of resources within the primary conidiophores. Kikuma at al. [56] showed that autophagy occurs during conidiophore development in *A. oryzae*, and they suggested that nutrients liberated in this manner are critical for conidiophore development. Considering the intense vacuolization we find in the embedded conidiophores of *A. fumigatus* (Figure 1A), it seems likely that autophagy is also occurring here even though it is immersed in a nutrient-rich medium. Studies about developmental flexibility and autophagy in conidiophores are now in progress.

### Summary and implications

In summary, we discovered that oxygen is a critical environmental factor for proper activation of *AfubrlA* and conidiogenesis, from which the signal proceeds via the upstream regulators (Flbs) in aspergilli (at least in *A. fumigatus* AF293). Indeed, *A. fumigatus* AF293 recognizes the phase transition (solid to air) as an extracellular cue for de-repression of *AfubrlA* and *AfuabaA* expression and the progression of conidiogenesis, separately from the oxygen-mediated upstream pathway. These results greatly enhance our understanding of the signals that govern the regulatory pathways of conidiogenesis in *A. fumigatus* and strongly suggest that investigations to identify genetic elements involved in both oxygen and phase-transition sensing are further required.

## Supporting Information

Figure S1 **Culture methods for microscopic observation of colony development in media-air interface.** (A) Cellophane covering method. Inoculums of fungal strains (small agar block containing active growing mycelia or conidial suspension) were covered with sterile cellophane membrane and cultured for 3–7 days at 30°C. Oxygen concentration was manipulated by putting the plate in a Ziploc® bag filled with ambient air, oxygen, or nitrogen gas. (B) Colony sectioning method. A few microliters of conidial suspension (5×10^6^ conidia/ml) of fungal strains was inoculated on GMM agar plates and cultured for 3–4 days at the appropriate temperature (25, 30 or 37°C).Grown colony was dissected vertically along the colony diameter, and thin sections of the agar blocks containing mycelia were stained with dye and/or applied for light microscopy. (C) Slide culture method. Conidial suspensions of the fungal strains were streaked on GMM agar media and the agar block was sliced into the thin sections. The thin agar blocks were sandwiched between plastic cover slips and glass slides. The agar blocks were cultured in humid chamber at 30°C for 1–5 days. (D) Sandwiched culture method. Conidia of the fungal strains were inoculated on center of the thin agar blocks using sterile cotton swabs, and the agar blocks were sandwiched between plastic cover slips and glass slides. Note that the inoculum is located in the gel-embedded environment.
(PDF)

Figure S2 **Conidiophore development of various aspergilli in response of oxygen and aeriform environment.** Twenty aspergilli were inoculated on PDA and cultured in high or low oxygen condition with or without cellophane membrane cover on the colony. Pictures were taken at 60 hours after inoculation. Note that this is pictorial replication of Table 1. Bars  =  200 µm.
(PDF)

Table S1 **Aspergillus strains used in this study.** a) Two AF293 from different depositor was used for covering potential genetic variation. b) Fungal genetics stock center c and d) These strains were provided from Dr. Cramer in Montana State University, USA, which were originally collected by Dr. Paul Dyer (University of Nottingham, UK) and Dr. Jean-Paul Latgé (Institut Pasteur, France). e) These strains were provided from Dr. Jae-Hyuk Yu in University of Wisconsin-Madison, USA. f) ARS (Agricultural Research Service) culture collection, USDA, USA.
(PDF)

Table S2 **Evaluation of conidiophore development in gel-embedded environment in different** ***A. fumigatus*** **strains.** a) Two AF293 from different depositor was used for covering potential genetic variation.
(PDF)

Table S3 **Primer used in this study.**
(PDF)
