# Supplementary material for: Oxygen and an Extracellular Phase Transition Independently Control Central Regulatory Genes and Conidiogenesis in Aspergillus fumigatus
Source: PLoS One. 2013 Sep 5;8(9):e74805. doi: 10.1371/journal.pone.0074805 (PMC3764054; doi:10.1371/journal.pone.0074805)

Figure S1.

A. Cellophane covering method

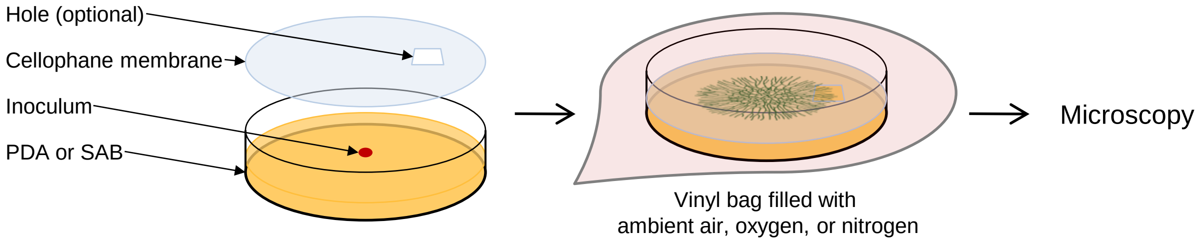

B. Colony sectioning method

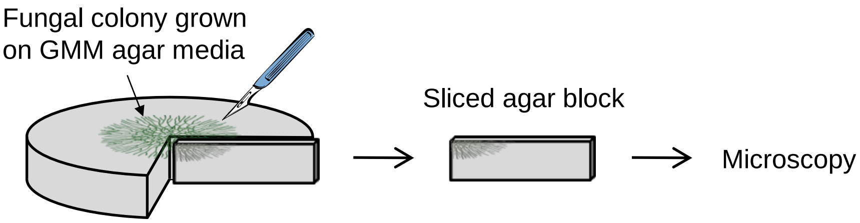

C. Slide culture method

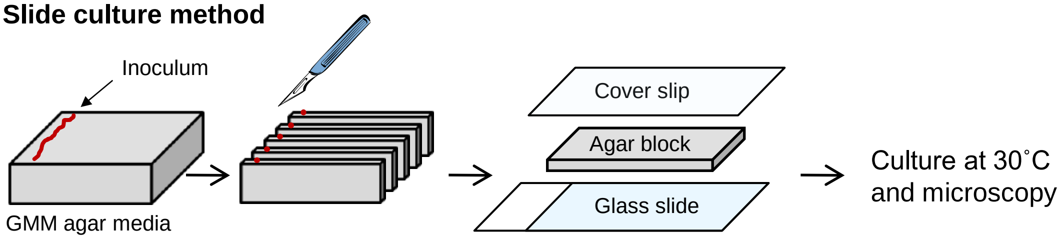

D. Sandwiched culture method

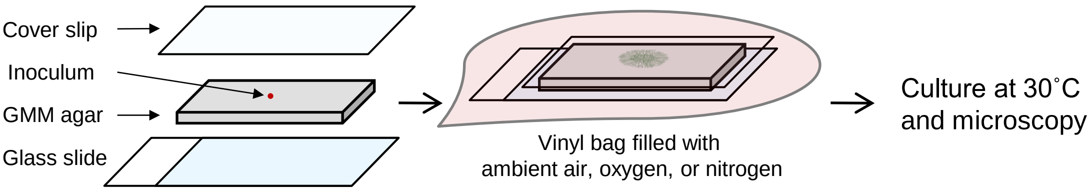

Supplement: Figure S1 — Culture methods for microscopic observation of colony development in media-air interface. (A) Cellophane covering method. Inoculums of fungal strains (small agar block containing active growing mycelia or conidial suspension) were covered with sterile cellophane membrane and cultured for 3–7 days at 30°C. Oxygen concentration was manipulated by putting the plate in a Ziploc® bag filled with ambient air, oxygen, or nitrogen gas. (B) Colony sectioning method. A few microliters of conidial suspension (5×10^6^ conidia/ml) of fungal strains was inoculated on GMM agar plates and cultured for 3–4 days at the appropriate temperature (25, 30 or 37°C).Grown colony was dissected vertically along the colony diameter, and thin sections of the agar blocks containing mycelia were stained with dye and/or applied for light microscopy. (C) Slide culture method. Conidial suspensions of the fungal strains were streaked on GMM agar media and the agar block was sliced into the thin sections. The thin agar blocks were sandwiched between plastic cover slips and glass slides. The agar blocks were cultured in humid chamber at 30°C for 1–5 days. (D) Sandwiched culture method. Conidia of the fungal strains were inoculated on center of the thin agar blocks using sterile cotton swabs, and the agar blocks were sandwiched between plastic cover slips and glass slides. Note that the inoculum is located in the gel-embedded environment. (PDF) [file pone.0074805.s001.pdf]
