# Supplementary material for: Oxygen and an Extracellular Phase Transition Independently Control Central Regulatory Genes and Conidiogenesis in Aspergillus fumigatus
Source: PLoS One. 2013 Sep 5;8(9):e74805. doi: 10.1371/journal.pone.0074805 (PMC3764054; doi:10.1371/journal.pone.0074805)
Supplement: Figure S2 — Conidiophore development of various aspergilli in response of oxygen and aeriform environment. Twenty aspergilli were inoculated on PDA and cultured in high or low oxygen condition with or without cellophane membrane cover on the colony. Pictures were taken at 60 hours after inoculation. Note that this is pictorial replication of Table 1. Bars = 200 µm. (PDF) [file pone.0074805.s002.pdf]

Figure S2.

Species name	Strain ID	Aeriform		Gel-phase		Type	Section
		High-oxygen	Low-oxygen	High-oxygen	Low-oxygen		
Aseprgillus fumigatus	FGSC A1100	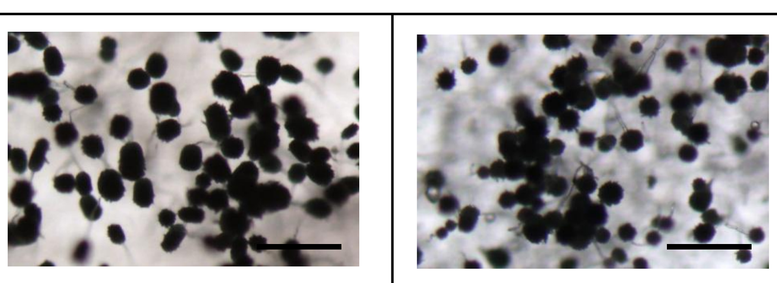		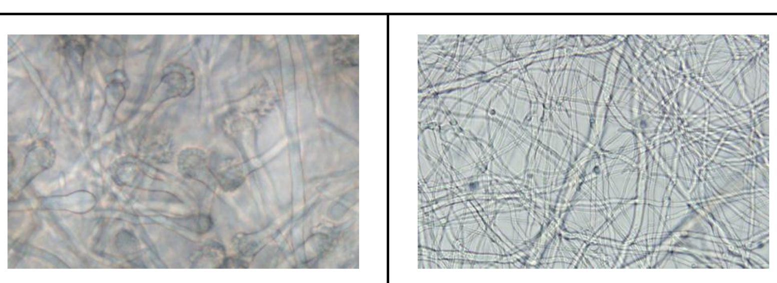		A	Fumigati
Aspergillus parvulus	NRRL 2667	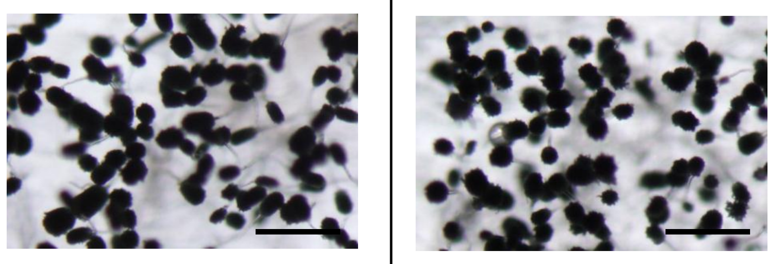		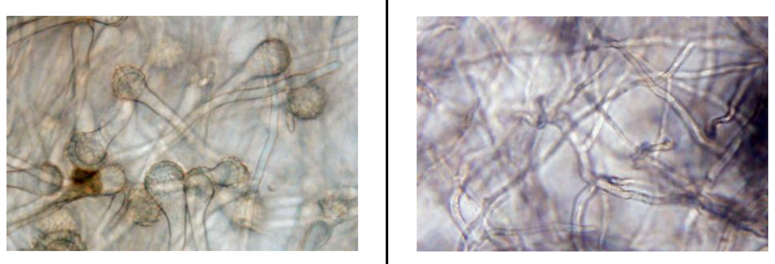		A	Cervini
Aspergillus kanagawaensis	NRRL 5023	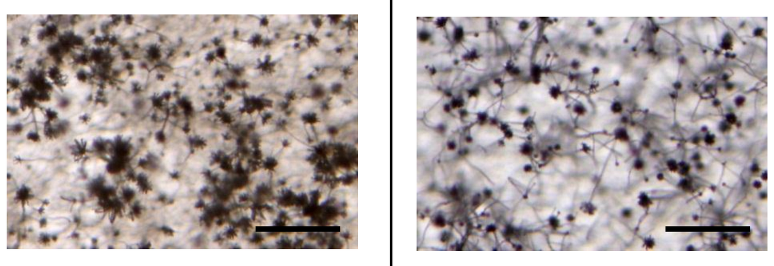		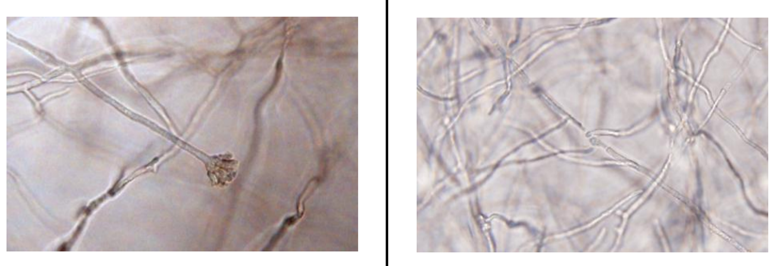		A	Cervini
Aspergillus nidulans	FGSC A117	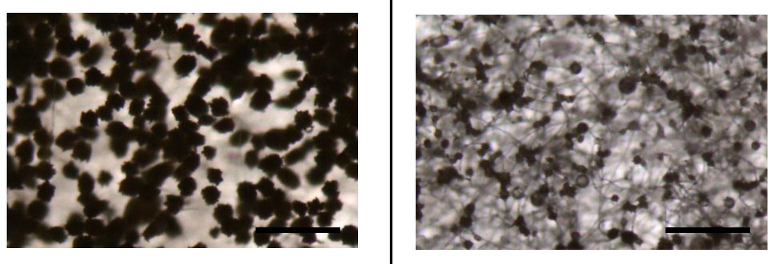		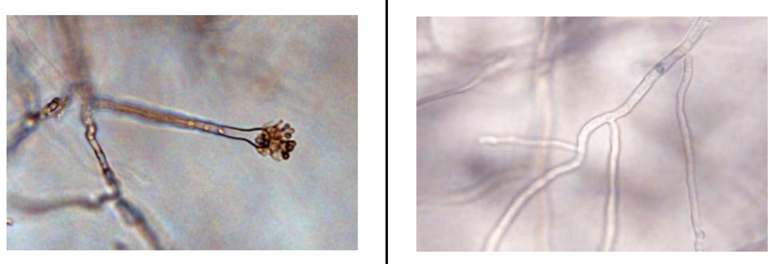		A	Nidulantes
Aspergillus unilateralis	NRRL 577	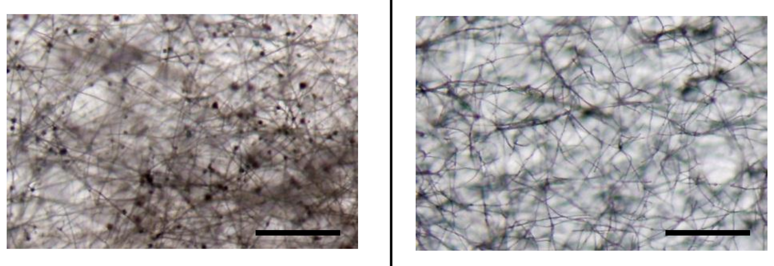		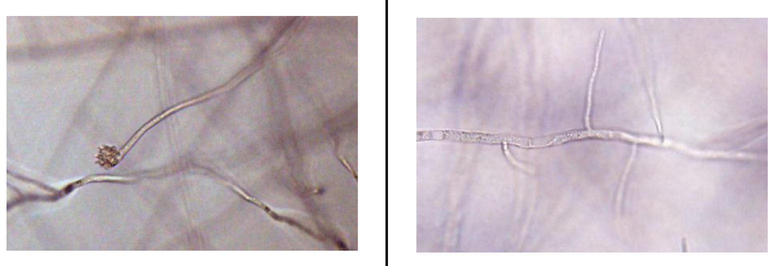		B	Fumigati
Aspergillus niger	FGSC A732	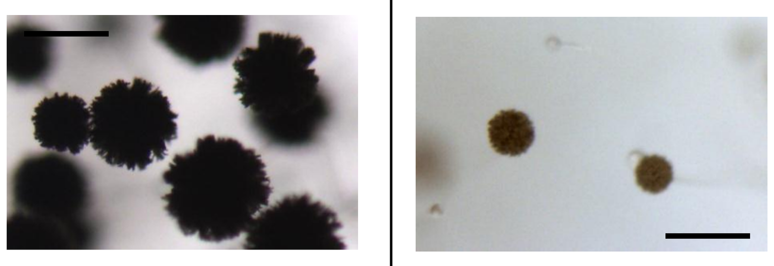		Data not shown	Data not shown	C	Nigri
Aspergillus nidulans	FGSC A90	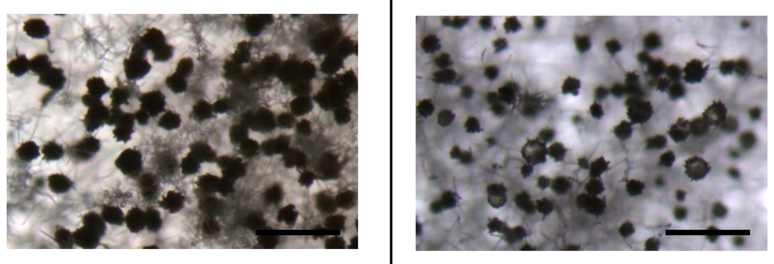		Data not shown	Data not shown	C	Nidulantes
Aspergillus awamori	FGSC A808	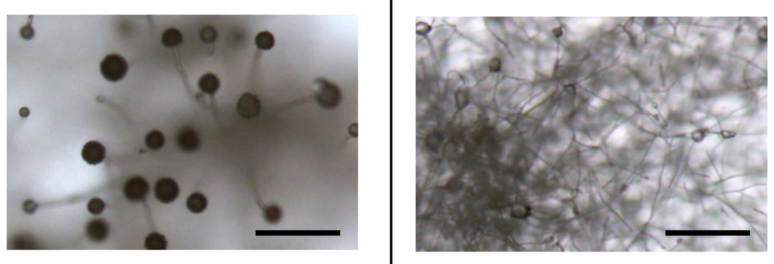		Data not shown	Data not shown	D	Nigri
Aspergillus terreus	FGSC A1156	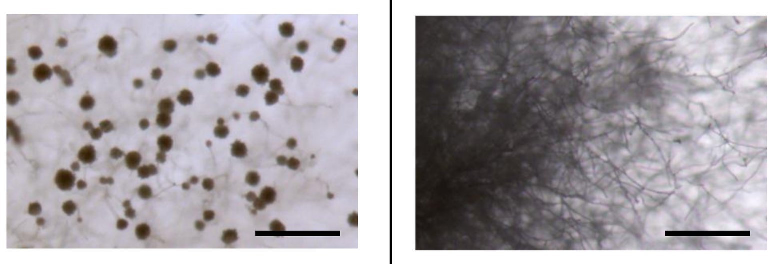		Data not shown	Data not shown	D	Terrei
Aspergillus terreus	NRRL 260	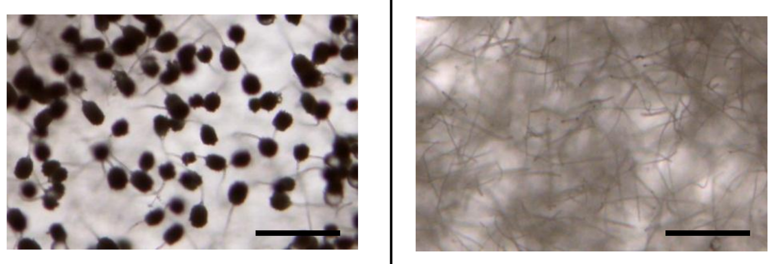		Data not shown	Data not shown	D	Terrei
Aspergillus oryzae	FGSC A815	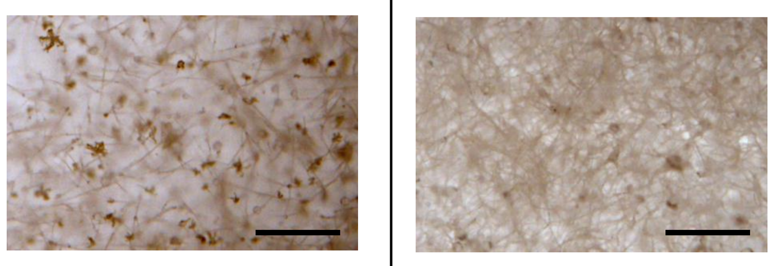		Data not shown	Data not shown	D	Flavi
Aspergillus nidulans	FGSC A4	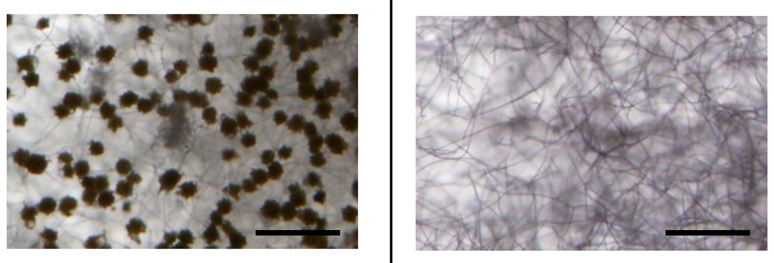		Data not shown	Data not shown	D	Nidulantes
Aspergillus giganteus	NRRL 10	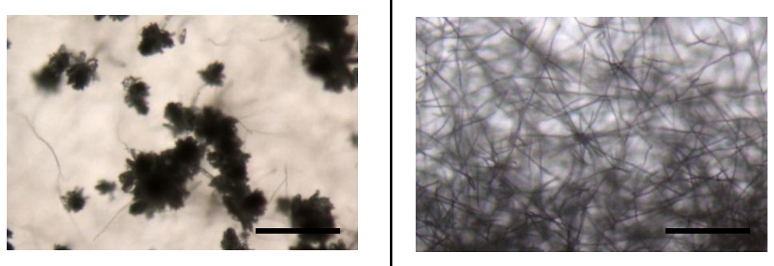		Data not shown	Data not shown	D	Clavati
Aspergillus clavatus	NRRL 1	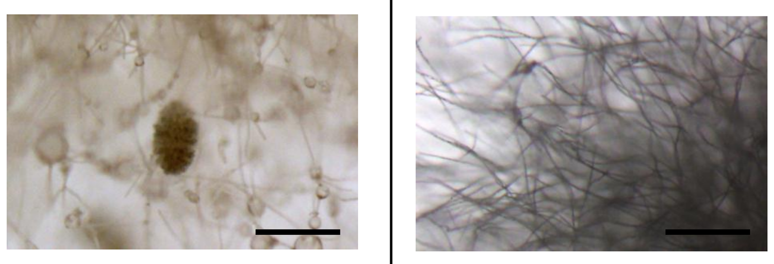		Data not shown	Data not shown	D	Clavati
Neosartorya pseudofischeri	NRRL 20748	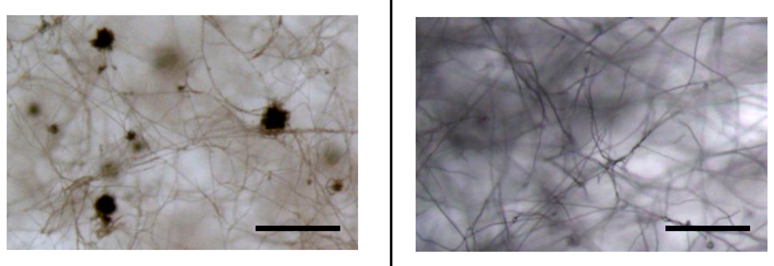		Data not shown	Data not shown	D	Fumigati
Aspergillus heterothallicus	FGSC A251	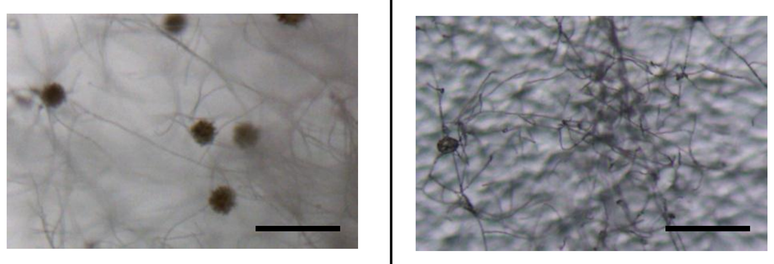		Data not shown	Data not shown	D'	Usti
Neocarpenteles acanthosporum	NRRL 5293	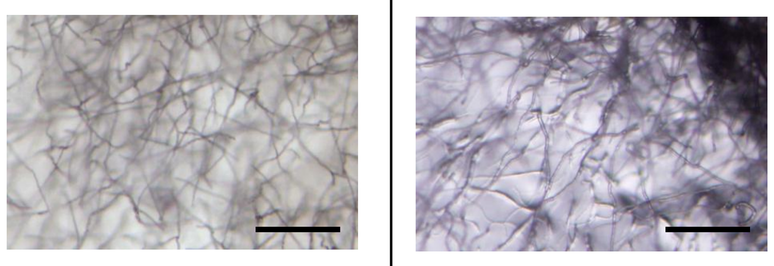		Data not shown	Data not shown	E	Clavati
Neosartorya aureola	NRRL 2244	Pictures not taken	Pictures not taken	Pictures not taken	Pictures not taken	E	Fumigati
Neosatorya fischeri	NRRL 181	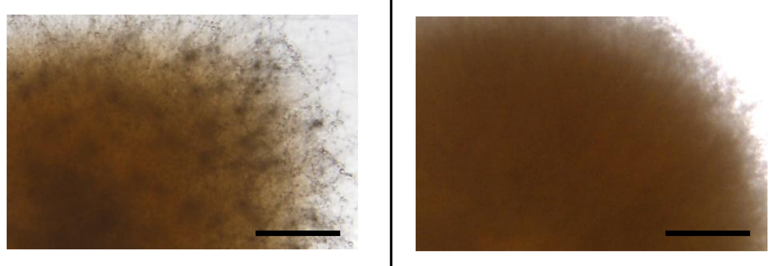		Data not shown	Data not shown	E	Fumigati
Dichotomomyces cejpii	NRRL 26980	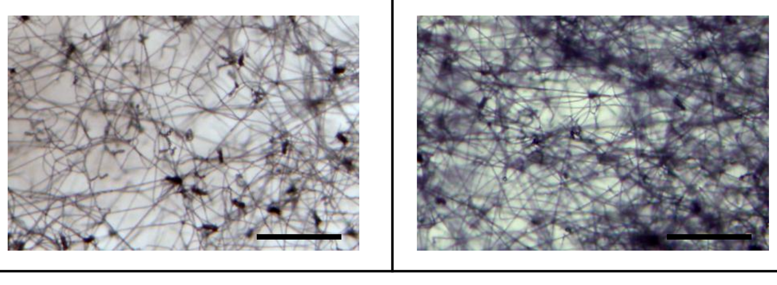		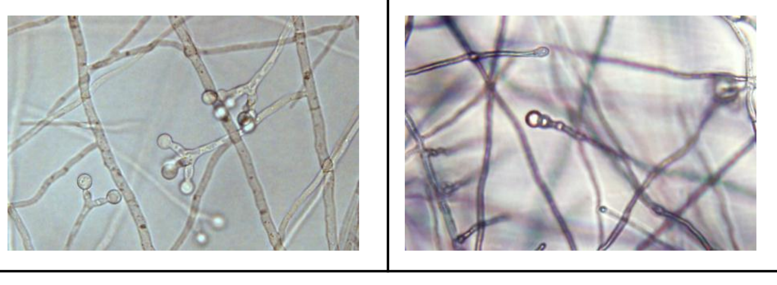		E'	Clavati
