# Supplementary material for: Oxygen and an Extracellular Phase Transition Independently Control Central Regulatory Genes and Conidiogenesis in Aspergillus fumigatus
Source: PLoS One. 2013 Sep 5;8(9):e74805. doi: 10.1371/journal.pone.0074805 (PMC3764054; doi:10.1371/journal.pone.0074805)
Supplement: Table S1 — Aspergillus strains used in this study. a) Two AF293 from different depositor was used for covering potential genetic variation. b) Fungal genetics stock center c and d) These strains were provided from Dr. Cramer in Montana State University, USA, which were originally collected by Dr. Paul Dyer (University of Nottingham, UK) and Dr. Jean-Paul Latgé (Institut Pasteur, France). e) These strains were provided from Dr. Jae-Hyuk Yu in University of Wisconsin-Madison, USA. f) ARS (Agricultural Research Service) culture collection, USDA, USA. (PDF) [file pone.0074805.s003.pdf]

Table S1. Aspergillus strains used in this study.

Strain name	Relevant genotype/information	Source	Reference
<*A. fumigatus* wild type strains>			
AF293 (A1100) [a]	*A. fumigatus* clinical isolate	FGSC [b]	[31,32,33]
AF293 (A1435) [a]	*A. fumigatus* clinical isolate	FGSC	[31,32,33]
AF10 (A1260)	*A. fumigatus* clinical isolate	FGSC	[33,34,35,36]
AF210 (A1259)	*A. fumigatus* clinical isolate	FGSC	[33,35,37]
AF1	*A. fumigatus* clinical isolate	Paul Dyer [c]	[34,35,36]
AF62	*A. fumigatus* environmental isolate	Paul Dyer	[34,35,36]
AF217	*A. fumigatus* environmental isolate	Paul Dyer	[36]
AF221	*A. fumigatus* environmental isolate	Paul Dyer	[36]
AF250	*A. fumigatus* clinical isolate	Paul Dyer	[36]
AFIR957	*A. fumigatus* environmental isolate	Paul Dyer	[40]
AFRB3	*A. fumigatus* environmental isolate	Paul Dyer	[40]
AF41	*A. fumigatus* clinical isolate	Paul Dyer	[34,36,41]
AF70	*A. fumigatus* environmental isolate	Paul Dyer	[34,35,36]
CBS144.89	*A. fumigatus* clinical isolate	Jean-Paul Latgé [d]	[42,43]
CBS133.61	*A. fumigatus* clinical isolate	Jean-Paul Latgé	[44]
<*A. fumigatus* mutant strains defective in conidiogenesis>			
ΔAf*fluG1*	Af*pyrG*1; ΔAf*fluG*::Af*pyrG*$^{+}$	J. H. Yu [e]	[27]
ΔAf*flbA4*	Af*pyrG*1; ΔAf*flbA*::Af*pyrG*$^{+}$	J. H. Yu	[27]
A1176	Af*pyrG*1; ΔAf*brlA*::Af*pyrG*$^{+}$	J. H. Yu	[27]
TKSS1.01	Af*pyrG*1; ΔAf*flbB*::Af*pyrG*$^{+}$	J. H. Yu	[30]
ΔAf*flbC*	Af*pyrG*1; ΔAf*flbC*::Af*pyrG*$^{+}$	J. H. Yu	Not published
TKSS6.07	ΔAfu*flbE*::Ani*pyrG*$^{+}$;Af*pyrG*1	J. H. Yu	[22]
TSGa17	Af*pyrG*1; ΔAfu*abaA*::Ani*pyrG*$^{+}$	J. H. Yu	[28]
TSGw4	Af*pyrG*1; ΔAfu*wetA*::Ani*pyrG*$^{+}$	J. H. Yu	[28]
THSF1.1	ΔAfu*veA*::Ani*pyrG*$^{+}$;Af*pyrG*1	J. H. Yu	[29]
THSF2.1	ΔAfu*velB*::Ani*pyrG*$^{+}$;Af*pyrG*1	J. H. Yu	[29]
TNI17.1	Af*pyrG*1; ΔAf*vosA*::Ani*pyrG*$^{+}$	J. H. Yu	[29]

<The other Aspergilli species>

A4	*A. nidulans* Glasgow wild type (veA$^+$)	FGSC	[33]
A90	*A. nidulans* Grindle wild isolate 2	FGSC	[33]
A117	*A. nidulans* Prototroph, wild type color (no veA$^+$)	FGSC	[33]
A251	*A. heterothallicus* wild type	FGSC	[33]
A732	*A. niger* wild type	FGSC	[33]
A815	*A. oryzae* wild type	FGSC	[33]
A1156	*A. terreus* wild type	FGSC	[33]
NRRL1	*A. clavatus* wild type	NRRL[f)]	[45]
NRRL10	*A. giganteus* wild type	NRRL	[45]
NRRL181	*Neosatorya fischeri* wild type	NRRL	[45]
NRRL20748	*Neosartorya pseudofischeri* wild type	NRRL	[45]
NRRL2244	*Neosartorya aureola* wild type	NRRL	[45]
NRRL260	*A. terreus* wild type	NRRL	[45]
NRRL2667	*A. parvulus* wild type	NRRL	[45]
NRRL26980	*Dichotomomyces cejpii* wild type	NRRL	[45]
NRRL5023	*A. kanagawaensis* wild type	NRRL	[45]
NRRL5293	*Neocarpenteles acanthosporum* wild type	NRRL	[45]
NRRL577	*A. unilateralis* wild type	NRRL	[45]
