# Supplementary material for: Oxygen and an Extracellular Phase Transition Independently Control Central Regulatory Genes and Conidiogenesis in Aspergillus fumigatus
Source: PLoS One. 2013 Sep 5;8(9):e74805. doi: 10.1371/journal.pone.0074805 (PMC3764054; doi:10.1371/journal.pone.0074805)
Supplement: Table S2 — Evaluation of conidiophore development in gel-embedded environment in different A. fumigatus strains. a) Two AF293 from different depositor was used for covering potential genetic variation. (PDF) [file pone.0074805.s004.pdf]

Table S2. Evaluation of conidiophore development in gel-embedded environment in different *A. fumigatus* strains.

Strain Name	Clinical/ Environmental	Mating type	Origin	Conidiophores in gel-phase
AF1	C	Mat 1-1	USA	Produced
AF62	E	Mat 1-1	USA	Produced
AF217	E	Mat 1-1	Sweden	Produced
AF250	C	Mat 1-1	UK	Produced
AFIR957	E	Mat 1-1	Ireland	Produced
AFRB3	E	Mat 1-2	Ireland	Produced
AF221	E	Mat 1-2	New Zealand	Produced
AF293 (A1100)[a)]	C	Mat 1-2	UK	Produced
AF293 (A1435)[a)]	C	Mat 1-2	UK	Produced
AF210	C	Mat 1-1	UK	Not produced
CBS144.89	C	Mat 1-1	France	Not produced
CBS133.61	C	Mat 1-1	USA	Not produced
AF10	C	Mat 1-2	USA	Not produced
AF41	C	Mat 1-2	USA	Not produced
AF70	E	Mat 1-2	USA	Not produced
