# Supplementary material for: Oxygen and an Extracellular Phase Transition Independently Control Central Regulatory Genes and Conidiogenesis in Aspergillus fumigatus
Source: PLoS One. 2013 Sep 5;8(9):e74805. doi: 10.1371/journal.pone.0074805 (PMC3764054; doi:10.1371/journal.pone.0074805)
Supplement: Table S3 — Primer used in this study. (PDF) [file pone.0074805.s005.pdf]

Table S3. Primer used in this study.

Primer Name	Sequence (5’ to 3’)	Description
Af_Btub_F	ATAATGTTCAGACCGCCCTCTGCT	Forward primer for β-tubulin
Af_Btub_R	GACGGATGTGGAATTGCCCACAAA	Reverse primer for β-tubulin
Afu_BrlA_RTF	ACCTACCCCTACGAGCATCT	Forward primer for *AfubrlA*
Afu_BrlA_RTR	TCATACGAAGGCAGGAAGTC	Reverse primer for *AfubrlA*
Afu_AbaA_RTF	GCAGTTCTTCCGCTCTTTAT	Forward primer for *AfuabaA*
Afu_AbaA_RTR	GGCTTCGGACTATTCATCAT	Reverse primer for *AfuabaA*
Afu_WetA_RTR	CTCGCGTCTTGCTTTAGTCT	Forward primer for *AfuwetA*
Afu_WetA_RTF	CACAAAGACTAGCCCTCACC	Reverse primer for *AfuwetA*
